# A novel model of cardiovascular–kidney–metabolic syndrome combining unilateral nephrectomy and high-salt–sugar–fat diet in mice

**DOI:** 10.1038/s41684-024-01457-5

**Published:** 2024-10-22

**Authors:** Lucas Rannier R. A. Carvalho, Miho Shimari, Ariela Maína Boeder, Zhengbing Zhuge, Min Cai, Cecilia Leijding, Stefano Gastaldello, Andrei L. Kleschyov, Tomas A. Schiffer, Drielle Dantas Guimarães, Gaia Picozzi, Lars H. Lund, Bengt Fellström, Eddie Weitzberg, Jon O. Lundberg, Carolina E. Hagberg, Gianluigi Pironti, Daniel C. Andersson, Mattias Carlström

**Affiliations:** 1 Department of Physiology and Pharmacology, Karolinska Institutet, Stockholm, Sweden; 2 Department of Pharmacology, Federal University of Santa Catarina, Florianopolis, Brazil; 3 Division of Cardiovascular Medicine, Department of Medicine Solna, Karolinska Institutet, Stockholm, Sweden; 4 Center for Molecular Medicine, Karolinska Institutet, Stockholm, Sweden; 5 Department of Medicine, Cardiology Unit, Karolinska Institutet, Stockholm, Sweden; 6 Department of Cardiology, Karolinska University Hospital, Stockholm, Sweden; 7 Department of Medical Science, Renal Unit, University Hospital, Uppsala, Sweden; 8 Heart, Vascular and Neurology Theme, Cardiology Unit, Karolinska University Hospital, Stockholm, Sweden

**Keywords:** Chronic kidney disease, Hypertension, Metabolic syndrome

## Abstract

The aim of this study was to explore biological interaction and pathophysiology mechanisms in a new mouse model of cardiovascular–kidney–metabolic (CKM) syndrome, induced by chronic moderate renal failure in combination with consumption of a customized Western diet rich in carbohydrates, fat and salt. Male C57BL/6J mice were subjected to unilateral nephrectomy, fed a customized Western diet rich not only in sugar and fat but also in salt, and followed for 12 weeks or 20 weeks. Sham-operated mice on a standard chow served as healthy controls. Body composition, weight gain, glucose metabolism, fat distribution, blood pressure, cardiac function, vascular reactivity, renal function, inflammation and mitochondrial function were measured and combined with biochemical and histopathological analyses. The novel triple-hit model of CKM syndrome showed signs and symptoms of metabolic syndrome, disturbed glucose metabolism, impaired adipocyte physiology and fat redistribution, cardiovascular dysfunction, renal damage and dysfunction, systemic inflammation, elevated blood pressure and cardiac remodeling. The pathological changes were more pronounced in mice after prolonged exposure for 20 weeks, but no deaths occurred. In the present mouse model of CKM syndrome, profound and significant metabolic, cardiac, vascular and renal dysfunctions and injuries emerged by using a Western diet rich not only in fat and carbohydrates but also in salt. This multisystem disease model could be used for mechanistic studies and the evaluation of new therapeutic strategies.

## Abstract (web summary)

The authors describe a novel mouse model of cardiovascular–kidney–metabolic syndrome induced by unilateral nephrectomy and a Western diet rich in carbohydrates, fat and salt, which could be used to study human condition and identify therapies.

## Main

Cardiovascular disease (CVD), chronic kidney disease (CKD) and type 2 diabetes (T2D) are among the most disruptive public health issues of the twenty-first century. Taken individually, each of these three conditions is associated with elevated morbidity and mortality and represents one of the biggest concerns of preventive health systems in the world^1^. In numbers, currently more than 800 million people suffer from CKD worldwide^2^, and there are about 65 million persons diagnosed with heart failure^3,4^. Moreover, accumulating research shows that hypertension is a major modifiable risk factor for CVD (including coronary artery disease, heart failure and stroke) and CKD^5^. This is a major problem considering that hypertension and its associated complications are affecting over one billion people worldwide^6^. Regarding diabetes, one in ten people is estimated to live with diabetes, the majority with T2D, according to the International Diabetes Federation. Notably, it is predicted that the number of patients with diabetes—currently 550 million—will rise to 643 million by 2030 and 783 million by 2045^4^. Among the modifiable risk factors for developing CVD (including hypertension) and T2D, our modern lifestyle, characterized by too little exercise and unhealthy diets leading to overweight and obesity, is particularly critical^7,8^.

In the past few years, the major overlap between the pathophysiological mechanisms of these diseases^9,10^, as well as their joint effects, which can be easily observed epidemiologically, have been the topic of many discussions. For example, CKD prevalence is three times higher in patients with T2D, and 50% higher among individuals with heart failure^11^. Furthermore, T2D is associated with a two- to four-fold higher risk of developing CVD^12^. The integrated yet independent pathological changes in the cardiovascular, renal and metabolic systems, recently defined by the American Heart Association as the cardiovascular–kidney–metabolic (CKM) syndrome^13^, constitute a condition that can be diagnosed without a defined primary cause, particularly in advanced cases. In addition, even with the growing body of evidence demonstrating the strong interplay between T2D, CVD and CKD, some of the mechanisms are still unknown. There are many descriptions of the effects of T2D as a risk factor for CVD and CKD; however, the opposite is little explored. Indeed, how cardiovascular–renal pathological changes can reflect on the metabolic phenotype, initiation and control of T2D are less known^1^.

Clinical and epidemiological studies provide ample information regarding the prognosis, diagnostic methods, biomarkers and treatments of CKM disorders, which are rapidly increasing worldwide^9,14^. Yet, mechanistic investigations on CKM syndrome are scarce. To our knowledge, there is no in vitro approach that reproduces the changes observed in CKM syndrome. The in vitro models using spheroids to study specific and isolated systems show great potential^15–17^, yet cannot integrate multiorgan systems such as CKM syndrome. The multitude of possible interacting mechanisms in the development and progression of CKM syndrome, including perturbations in cardiovascular, renal and metabolic functions, necessitates an in vivo model that integrates all these systems.

Most animal models currently used focus on metabolic, cardiovascular or renal dysfunction rather than on the combination of all. Even in mammalian models, the simultaneous induction of pathological alterations in the three systems for the development of CKM syndrome has had limited success. Inducing multisystemic changes indirectly and with low mortality rate is a critical hurdle in current animal models of CKM syndrome. Therefore, most studies suppress the metabolic pillar and focus only on the cardiorenal complex, regardless of whether the phenotype is acute or chronic^18–20^.

Facing the need for new models and mechanistic options to investigate CKM syndrome and given the difficulty in reproducing clinically relevant pathological changes in all three systems, the aim of this work was to characterize a new model of CKM syndrome in mice. Here, we combined early-life unilateral nephrectomy (UNX) with chronic consumption of a customized Western diet (WD) rich not only in sugar and fat but also in salt to induce kidney dysfunction, metabolic syndrome and hypertension. A schematic of the experimental design of the model is shown in Supplementary Fig. 1.

## Results

In vivo and ex vivo parameters associated with the renal, cardiovascular and metabolic parameters did not differ significantly between sham-operated mice fed regular chow for 12 and 20 weeks. Sham results presented in all the tables and figures have therefore been merged and compared with the mice exposed to UNX and high-salt WD treatment.

### Reduced nephron number and high-salt WD induces changes in the metabolic profile, but not in body weight

**Fig. 1:**
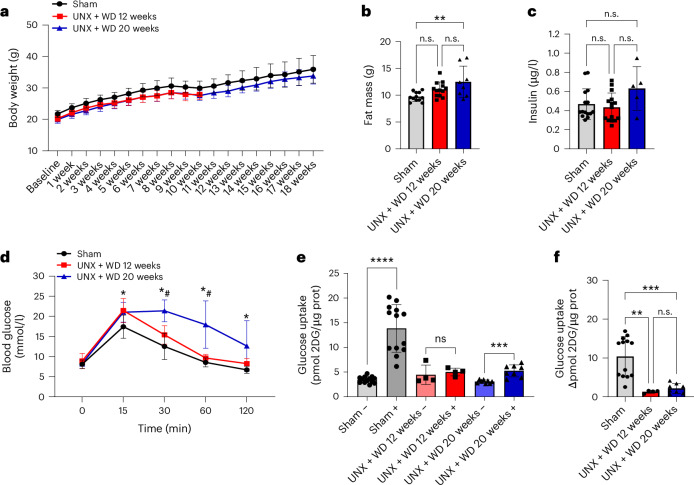
Metabolic parameters. Evaluation of the metabolic profile of mice with early-life UNX and consuming a modified WD with high sugar, fat and salt for 12 and 20 weeks. **a**, Body weight curve in grams. **b**, Fat mass quantified by dual-energy X-ray absorptiometry scan. **c**, Serum insulin levels in a fed state in the first hours of the day. **d**, Intraperitoneal glucose tolerance test (ipGTT); *, compared with sham group; ^#^, compared with UNX + WD 12 weeks group. **e**, Glucose uptake in isolated skeletal muscle (extensor digitorum longus) with (+) and without (−) insulin. **f**, The insulin-mediated glucose uptake presented as the change in glucose uptake with and without insulin in muscles from the same animal. 2DG, 2-deoxyglucose; prot, protein. The data are expressed as mean ± s.d., and the number of samples per method is described in Supplementary Table 2. For all parameters, one-way ANOVA was performed followed by Tukey’s multiple comparison test, except for the glucose tolerance test (**d**) where the two-way ANOVA test was performed. n.s., not significant, *P < 0.05, **P < 0.01, ***P < 0.001. ^#^, Compared with the UNX + WD 12 weeks group. Source data

Mice subjected to UNX at an early age (4 weeks old) and subsequently exposed to a customized WD, rich in fat and sugar together with 4% salt, did not display significant changes in body weight compared with controls, regardless of whether the mice were fed the diet for 12 or 20 weeks (Fig. 1a). This finding is similar to what was previously reported for the consumption of high-salt diet^21^. However, there was a significant increase in fat mass after 20 weeks of diet compared with sham mice (*P* < 0.05, one-way analysis of variance (ANOVA) and Tukey’s multiple comparison test), coupled with a reduction in lean mass at both time points (Fig. 1b and Table 1).

**Table 1:** Metabolic parameters in mice submitted to early-age UNX and fed with high-salt WD for 12 and 20 weeks, compared with the sham-operated mice on RD

Metabolic parameters	Sham + RD	UNX + WD 12 weeks	UNX + WD 20 weeks
Food intake (g/day)	3.98 ± 0.12	4.15 ± 0.27	4.26 ± 0.31
Water intake (ml/day)	4.47 ± 0.44	7.81 ± 0.67*	7.04 ± 0.23*
ipGTT AUC (a.u.)	1,192.0 ± 161.6	1,418 ± 105.9	2,042 ± 493.4*^#^
Fasting glucose (mmol/l)	7.60 ± 1.29	8.91 ± 1.90*	8.30 ± 0.72
Nonfasting glucose (mmol/l)	7.77 ± 1.04	9.36 ± 0.82*	7.71 ± 0.88^#^
Total mass (g)	32.75 ± 2.10	32.16 ± 1.66	34.27 ± 2.25
Fat percentage (%)	28.86 ± 3.96	34.40 ± 2.68*	36.72 ± 5.33*^#^
Lean mass (g)	22.57 ± 2.33	20.29 ± 0.62*	20.46 ± 1.60*
Lean percentage (%)	68.83 ± 4.11	63.19 ± 2.57*	61.82 ± 6.88*
Fat/lean mass ratio (a.u.)	0.42 ± 0.07	0.56 ± 0.06*	0.60 ± 0.18*
BMC (g)	0.79 ± 0.14	0.77 ± 0.11	0.70 ± 0.05
BMD (g/cm²)	0.073 ± 0.006	0.073 ± 0.005	0.071 ± 0.004
Plasma triglycerides (mg/dl)	131.21 ± 22.57	106.0 ± 54.15	118.6 ± 37.08
Plasma total cholesterol (mg/dl)	33.92 ± 9.15	44.08 ± 15.21*	47.81 ± 9.22*
Liver weight (g)	1.58 ± 0.21	1.32 ± 0.09*	1.34 ± 0.11*
Adipocyte area gWAT (µm²)	1,808.6 ± 449.89	2,538.3 ± 353.17*	2,420.3 ± 492.34*
Adipocyte area scWAT (µm²)	792.04 ± 108.88	1,577.9 ± 391.41*	1,125.1 ± 417.95^#^
*Cd68* mRNA expression gWAT (a.u.)	1.00 ± 0.46	0.96 ± 0.40	1.2 ± 1.64
*Jam2* mRNA expression gWAT (a.u.)	1.00 ± 0.12	0.61 ± 0.22**	0.69 ± 0.39
*Pdgfra* mRNA expression gWAT (a.u.)	1.00 ± 0.11	1.4 ± 0.32*	1.0 ± 0.53
*Lipe* mRNA expression gWAT (a.u.)	1.00 ± 0.26	0.83 ± 0.36	0.97 ± 0.72
*Lpl* mRNA expression gWAT (a.u.)	1.00 ± 0.22	0.70 ± 0.51	0.63 ± 0.46
*Ptprc* mRNA expression gWAT (a.u.)	1.0 ± 0.28	0.89 ± 0.21	1.2 ± 0.61
*Il1b* mRNA expression gWAT (a.u.)	1.0 ± 0.30	1.7 ± 0.83	1.4 ± 0.96
*Il6* mRNA expression gWAT (a.u.)	1.1 ± 0.56	0.87 ± 0.33	2.2 ± 2.0
*Ccl2* mRNA expression gWAT (a.u.)	1.1 ± 0.49	1.6 ± 0.85	1.7 ± 1.5

Values are expressed as mean ± s.d. a.u., arbitrary units; AUC, area under the curve; BMC, bone mineral content; BMD, bone mineral density; gWAT, gonadal white adipose tissue; scWAT, subcutaneous white adipose tissue. Sham + regular rodent chow (RD, *n* = 12); UNX + WD 12 weeks (*n* = 12); UNX + WD 20 weeks (*n* = 8). For all parameters, one-way ANOVA was performed followed by Tukey’s multiple comparison test. *, *P* < 0.05 compared with the corresponding sham group; **, *P* < 0.01 compared with the corresponding sham group. ^#^, *P* < 0.05 compared with the UNX + WD 12 weeks group.

Mice in the UNX + WD model group also had significantly higher fasting and nonfasting plasma glucose and insulin levels (*P* < 0.05, one-way ANOVA and Tukey’s multiple comparison test) (Table 1 and Fig. 1c). Moreover, the mice displayed disturbed glucose handling, as assessed by the in vivo glucose tolerance test (Fig. 1d) and ex vivo skeletal muscle insulin-mediated glucose uptake (Fig. 1e,f).

**Fig. 2:**
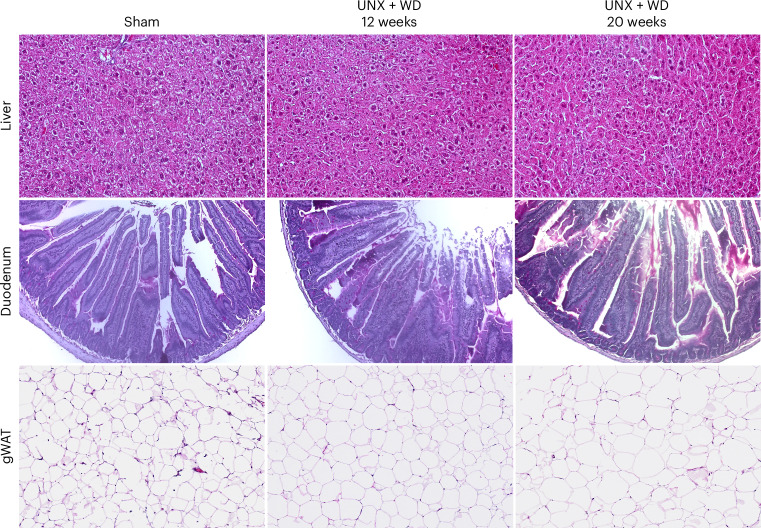
Histopathological evaluation of liver, duodenum and gWAT. Microscopic analyses of samples from mice subjected to UNX that consumed WD rich in salt for 12 and 20 weeks, compared with the control group. Samples stained with hematoxylin–eosin (HE). Top: photomicrographs of the liver parenchyma showing normal liver architecture, absence of inflammatory infiltrate and no signs of increased deposition of fat globules (20× objective). Middle: photomicrographs of the cross-section of the duodenal wall, to measure the size of the villi and crypts and evaluate the absorption surface and intestinal morphology (10× objective). Bottom: photomicrographs of gWAT with an evident increase in the average size of adipocytes in the groups exposed to UNX + WD compared with adipocytes from the same tissue in the sham-operated control group (20× objective).

Histopathological evaluation of the liver samples showed no differences in fat deposition between groups (Fig. 2). Results also showed a lower weight of the isolated liver from the UNX + WD groups compared with controls (Table 1), which is different from what has been commonly observed in animals exposed to WD, but in line with previous studies on high-salt diet‑induced metabolic changes^22,23^.

The UNX + WD mice displayed larger adipocytes in both subcutaneous and visceral fat, especially after 12 weeks of diet, despite no detectable difference in body weight (Table 1 and Fig. 2). The intestinal absorption area was also evaluated through duodenal histology; however, no significant changes were identified (Fig. 2). Further transcriptional analyses of the visceral adipose tissue indicated reduced tissue vascularization (assayed by *Jam2* expression), while relative expression of the preadipocyte marker *Pdgfra* was increased (Table 1) in the UNX + WD groups compared with the sham control group.

No other transcriptional markers, including those of immune cells or adipose tissue lipid handling, were significantly altered at the examined time points in the visceral adipose tissue, and no increase in immune cell infiltration was noted. Together, these findings suggest that the combined UNX + WD treatment causes a hypertrophied, poorly vascularized adipose tissue phenotype, potentially due to the high salt impairing preadipocyte differentiation, but without overt signs of adipose tissue inflammation (Table 1).

### Reduced nephron number and high-salt WD lead to the development of a CVD phenotype

**Fig. 3:**
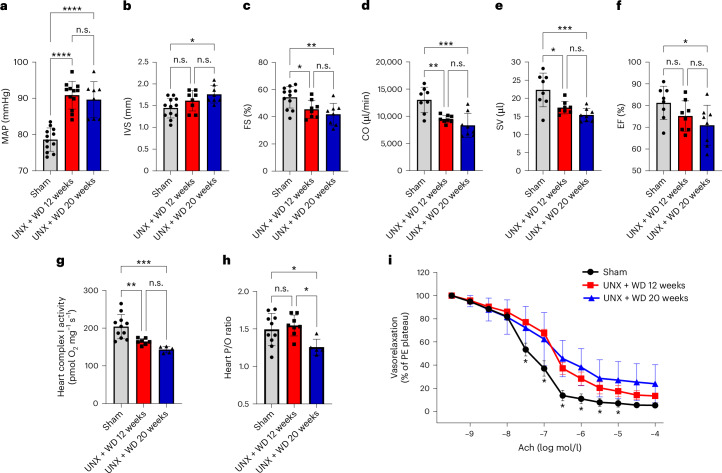
Cardiovascular functional parameters. The cardiovascular functional profile of mice that underwent UNX and were fed with a salt-rich WD for 12 and 20 weeks, compared with the sham-operated group consuming RD. **a**, MAP. **b**–**f**, Echocardiography investigation in anesthetized mice, to measure the following: IVS thickness (**b**); left ventricle FS (**c**); cardiac output (CO) (**d**); stroke volume (SV) (**e**); ejection fraction (EF) (**f**). **g**, The mitochondrial activity of heart complex I. **h**, The ratio for the number of atoms of phosphorus incorporated as ATP per molecule of oxygen (O_2_) consumed during oxidative phosphorylation in aerobically respiring cardiomyocytes mitochondria. **i**, The dose-dependent vessel relaxation curve of mesenteric arteries evaluated on myograph. PE, phenylephrine. The data are expressed as mean ± s.d., and the number of samples per method is described in Supplementary Table 2. For all parameters, one-way ANOVA was performed followed by Tukey’s multiple comparison test, with the exception of the Ach dose–response vessel relaxation curve (**i**) where a two-way ANOVA test was performed. *P < 0.05, ** *P* < 0.01, ***P < 0.001, **** *P* < 0.0001. Source data

Previous studies have shown that the chronic effects of salt consumption on the cardiovascular system extend well beyond increased blood pressure^24–26^. In the present novel CKM syndrome model, an increase of more than 10 mmHg in mean arterial pressure (MAP) was observed in the UNX + WD groups compared with the control group (Fig. 3a). Considering that these effects were observed in the C57BL/6J mouse strain, in which it is known to be difficult to induce a blood pressure increase, further exacerbated alterations are predictable in other more sensitive rodent strains^27^.

Heart function, evaluated with in vivo echocardiography, showed increased interventricular septum (IVS) diameter (Fig. 3b) and reduced left ventricular fractional shortening (FS), cardiac output and stroke volume as well as ejection fraction in the UNX + WD groups compared with the control group (Fig. 3c–f), which is consistent with impaired systolic function and hypertrophic remodeling commonly seen in long-term CVD (Table 2). In addition, reduced activity of mitochondrial complexes (I, II and IV) was observed as well as reduced efficiency (P/O ratio) in the heart mitochondria (Fig. 3g,h), especially after 20 weeks of WD (Table 2), indicating a myocardial functional decay.

**Table 2:** Cardiovascular parameters in mice submitted to early age UNX and fed with high-salt WD for 12 and 20 weeks, compared with the sham-operated mice on RD

Cardiovascular parameters	Sham + RD	UNX + WD 12 weeks	UNX + WD 20 weeks
SAP (mmHg)	96.16 ± 5.14	109.1 ± 3.51*	111.8 ± 5.02*
DAP (mmHg)	70.62 ± 2.56	82.12 ± 4.35*	84.39 ± 4.42*
HR (bpm)	582.1 ± 55.30	547.7 ± 37.59	534.7 ± 83.52
LVD (mm)	7.15 ± 0.72	6.71 ± 0.44	6.52 ± 0.29
LVS (mm)	3.50 ± 0.93	3.67 ± 0.64	3.88 ± 0.63
PW (mm)	1.84 ± 0.36	2.39 ± 0.52	2.76 ± 0.33*
*Nppb* (RQ)	1.14 ± 0.45	0.82 ± 0.18	0.85 ± 0.19
*Il1b* (RQ)	0.74 ± 0.22	0.87 ± 0.43	1.33 ± 0.53
Heart complex II activity (pmol O_2_ mg^−1^ s^−1^)	191.2 ± 28.72	160.7 ± 26.68	150.6 ± 11.56*
Heart complex IV activity (pmol O_2_ mg^−1^ s^−1^)	600.8 ± 74.47	520.3 ± 34.96*	534.5 ± 11.03*
DNIC signal (a.u.)	24.80 ± 3.72	29.80 ± 14.11	34.72 ± 16.88
Heme–NO signal (a.u.)	16.90 ± 3.26	10.36 ± 3.09*	4.55 ± 2.51*^#^
Nitrate plasma (μM)	19.78 ± 4.57	15.25 ± 3.37	17.63 ± 6.70
Nitrite plasma (μM)	0.60 ± 0.26	0.20 ± 0.07*	0.17 ± 0.20*

Values are expressed as mean ± s.d. DAP, diastolic arterial pressure; HR, heart rate; LVD, left ventricular diastolic dimension; LVS, left ventricular systolic dimension; PW, posterior wall thickness; RQ, relative quantification; SAP, systolic arterial pressure. Sham + RD (*n* = 12); UNX + WD 12 weeks (*n* = 12); and UNX + WD 20 weeks (*n* = 8). For all parameters, one-way ANOVA was performed followed by Tukey’s multiple comparison test. *, *P* < 0.05 compared with the corresponding sham group. ^#^, *P* < 0.05 compared with the UNX + WD 12 weeks group.

Vascular reactivity and endothelial function in isolated mesenteric resistance arteries were evaluated by ex vivo myography technique. Vessel segments from the UNX + WD model animals at 20 weeks displayed significantly reduced endothelium-dependent vasorelaxation (that is, responses to acetylcholine (Ach)) (*P* < 0.05, two-way ANOVA and Tukey’s multiple comparison test) (Fig. 3i). However, no significant differences were found in endothelium-independent relaxation induced by sodium nitroprusside (SNP) (data not shown).

**Fig. 4:**
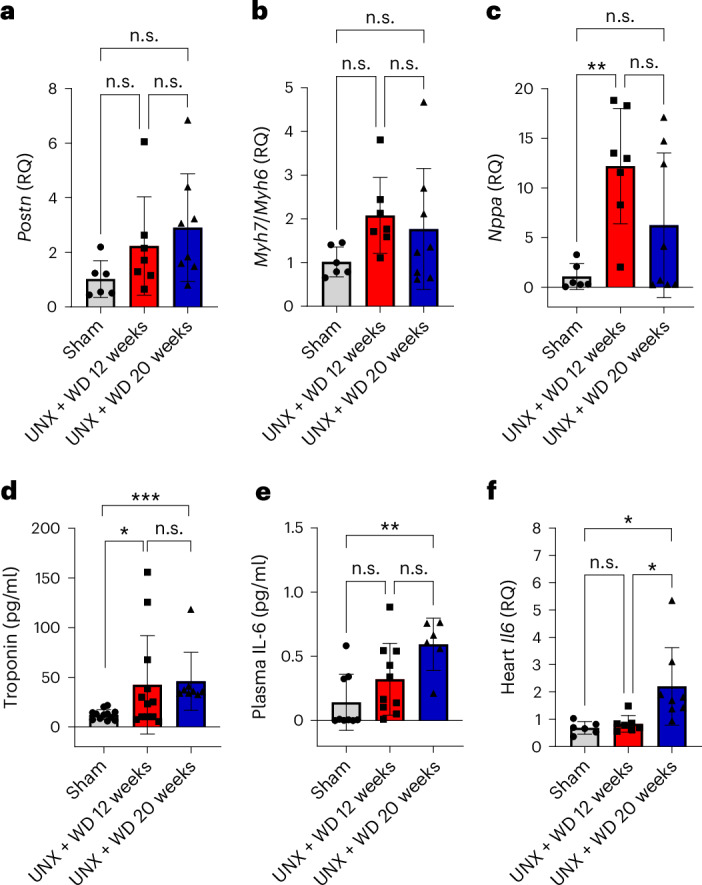
Cardiovascular biochemical and transcriptional parameters. The cardiovascular biochemical and transcriptional profile of mice that underwent UNX and were fed with a salt-rich WD for 12 and 20 weeks, compared with the sham-operated group consuming RD. mRNA or protein levels of biomarkers of cardiac injury, remodeling and inflammation were measured in cardiac tissue samples. **a**, *Postn*. **b**, The ratio of myosin heavy chain 7 (*Myh7*) and myosin heavy chain 6 (*Myh6*). **c**, ANP (*Nppa*). **d**, Troponin. **e**, IL-6 in plasma. **f**, *Il6* in cardiac tissue. The data are expressed as mean ± s.d. and relative quantification (RQ) of fold over control for mRNA data; the number of samples per method is described in Supplementary Table 2. For all parameters, one-way ANOVA was performed followed by Tukey’s multiple comparison test. *P < 0.05, **P < 0.01, ***P < 0.001. Source data

Atrial natriuretic peptide (ANP) and brain natriuretic peptide (BNP) are well-known cardiac stress markers. ANP (*Nppa*) expression level in the heart was significantly higher in the 12-week group compared with sham (*P* < 0.01, one-way ANOVA and Tukey’s multiple comparison test), whereas there was only a suggestive increase in the 20-week group indicating elevated cardiac stress (Fig. 4). On the contrary, the BNP (*Nppb*) level showed a slight decline in both UNX + WD groups compared with the sham group, although these differences were not statistically significant (Table 2). Despite not reaching a statistical significance, a slight incremental trend in the expression of periostin (*Postn*), a marker for active myofibroblast, and in the ratio of myosin heavy chain 7 to myosin heavy chain 6 (*Myh7*/*Myh6*, which refer to the fetal and the adult isoforms, respectively), suggested a cardiac adaptation in response to increased cardiac work in both CKM model groups (Fig. 4). The expression level of interleukin-1β (*Il1b*), primarily involved in the initial phase of inflammation, was not changed between groups (Table 2).

The elevation of biochemical markers of direct cardiac injury, including a three-fold increase in troponin levels between UNX + WD model groups and the control group, further indicates a substantial myocardial injury (Fig. 4d). Induction of interleukin-6 (IL-6)—a systemic inflammatory marker in the blood—was more pronounced at 20 weeks (Fig. 4e,f). Among the circulating markers of nitric oxide (NO) bioavailability, dinitrosyl iron complexes (DNIC), heme–NO and nitrite (but not nitrate) were significantly reduced in the model animals compared to controls (*P* < 0.05, one-way ANOVA and Tukey’s multiple comparison test), indicating reduced NO bioavailability in the mice with UNX + WD (Table 2).

**Fig. 5:**
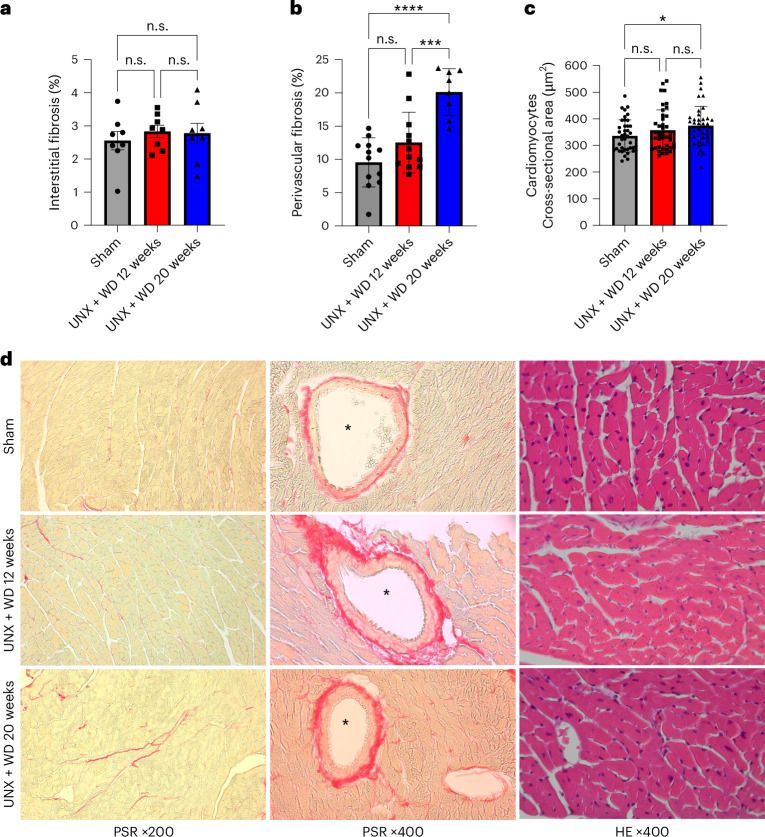
Histopathological evaluation of the heart. The cardiovascular histopathology of mice that underwent UNX and were fed with a salt-rich WD for 12 and 20 weeks, compared with the sham-operated group consuming RD. **a**, Interstitial fibrosis assessed by PicroSirius Red (PSR) staining at ×200 magnification (representative images in the first column in **d**). **b**, Perivascular fibrosis assessed by PSR staining at ×400 magnification (representative images in the second column in **d**). **c**, Cardiomyocyte size assessed by HE stain at ×400 magnification (representative images in the third column in **d**). The data are expressed as mean ± s.d., and the number of samples per method is described in Supplementary Table 2. For all parameters, one-way ANOVA was performed followed by Tukey’s multiple comparison test. *P < 0.05, **P < 0.01, ***P < 0.001, ****P < 0.0001. **d**, Representative photomicrographs of the experimental groups, staining techniques and magnifications used for histopathological evaluation. The asterisk indicates the lumen of the evaluated vessels. Source data

Finally, regarding the histopathological evaluation of the heart, the new model of CKM syndrome developed significantly increased perivascular fibrosis and cardiomyocyte size (UNX + WD 20 weeks versus sham; *P* < 0.05, one-way ANOVA and Tukey’s multiple comparison test) but without significant changes in interstitial fibrosis compared with the control group (Fig. 5a–d).

### Reduced nephron number and high-salt WD lead to structural damages and a decrease in kidney function

**Fig. 6:**
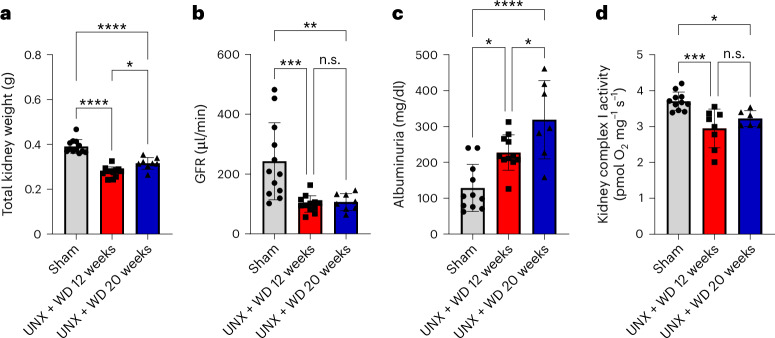
Renal parameters. Evaluation of the renal parameters of mice with UNX at young age and consuming a modified WD with high sugar, fat and salt for 12 and 20 weeks**. a**, The total weight of the kidneys in grams: sum of the weight of the two kidneys for the sham group and the weight of only the remaining right kidney for the UNX + WD groups. **b**, GFR assessed by inulin tail injection clearance. **c**, Urinary albumin concentration. **d**, Assessment of the activity of complex I of kidney mitochondria. The data are expressed as mean ± s.d., and the number of samples per method is described in Supplementary Table 2. For all parameters, one-way ANOVA was performed followed by Tukey’s multiple comparison test. *P < 0.05, **P < 0.01, ***P < 0.001, ****P < 0.0001. Source data

As expected, after 12 and 20 weeks, there was a compensatory increase in weight of almost 50% of the contralateral kidney (Fig. 6a). Despite this compensatory increase in kidney weight and a potential recruitment of more available nephrons, renal function was drastically impaired as revealed by the reduced glomerular filtration rate (GFR) and increased urinary albumin in both UNX + WD groups compared with the sham group (Table 3 and Fig. 6b,c). The renal mitochondrial function, assessed by the activity of complexes I, II and IV, was also impaired (Table 3 and Fig. 6d), especially after 12 weeks.

**Table 3:** Renal parameters in mice submitted to early age UNX and fed with high-salt WD for 12 and 20 weeks, compared with the sham-operated mice on RD

Renal parameters	Sham + RD	UNX + WD 12 weeks	UNX + WD 20 weeks
GFR:BW ratio (μl/min/g)	6.84 ± 3.06	3.09 ± 0.83*	3.94 ± 1.17*
Kidney complex II activity (pmol O_2_ µg^−1^ s^−1^)	6.05 ± 0.37	5.23 ± 0.94*	6.09 ± 0.62
Kidney complex IV activity (pmol O_2_ µg^−1^ s^−1^)	16.32 ± 0.72	14.05 ± 1.11*	16.64 ± 0.98^#^
Tubular injury score (0–10)	1.13 ± 0.94	6.53 ± 1.45*	8.00 ± 0.75*
Glomerular injury (%)	16.88 ± 3.53	45.05 ± 3.70*	58.72 ± 8.20*^#^
Glomerular area (µm^2^)	2,923 ± 766.1	4,425 ± 1,086*	6,414 ± 1,479*^#^
Fibrosis area (%)	3.08 ± 0.73	4.12 ± 1.68	6.05 ± 0.77*^#^

Values are expressed as mean ± s.d. BW, body weight. Sham + RD (*n* = 12); UNX + WD 12 weeks (*n* = 12); UNX + WD 20 weeks (*n* = 8). For all parameters, one-way ANOVA was performed followed by Tukey’s multiple comparison test. *P < 0.05 compared with the corresponding sham group. ^#^, *P* < 0.05 compared with the UNX + WD 12 weeks group.

**Fig. 7:**
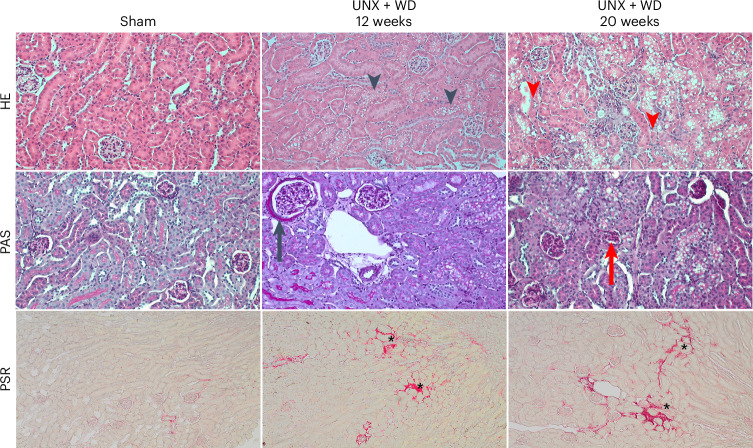
Histopathological evaluation of the kidney. Microscopic analyses of kidney samples from mice subjected to UNX that consumed WD rich in salt for 12 and 20 weeks, compared with the control group stained with three different methods. First line: HE staining with normal morphological appearance of the kidneys for the control group (sham) and signs of tubular injuries such as vacuolation (black arrowhead), degeneration and necrosis (red arrowhead) in the UNX + WD groups (20× objective). Second line: PAS staining of kidney samples from the sham group without evident histopathological changes, and from UNX + WD groups showing increased diameter of the glomerulus and thickening of the glomerular basement membrane (black arrow) and presence of glomerulosclerosis (red arrow) (20× objective). Third line: staining with PSR to highlight collagen fibers as an indicator of fibrosis; the positive areas in red were digitally identified for quantitative evaluation (*) (10× objective).

Histopathological evaluation was conducted in the same regions of the right kidneys in model and sham animals and with three different staining methods (Fig. 7). With all three methods, the control group did not show morphological changes or signs of cellular injury, as expected. By contrast, the model animals showed substantial tubular changes, including tubular vacuolization, dilatation and atrophy, loss of brusher border and thickening of tubular basement membrane (Fig. 7). The quantitative comparison of tubular and glomerular injuries, glomerular area and extent of fibrosis between the groups was performed using the specific scoring system (Table 3).

Evaluation of the glomeruli using periodic acid–Schiff (PAS) staining revealed an increase in the average diameter of the glomeruli in the model animals (Table 3 and Fig. 7) as well as an increased presence of glomerulosclerosis, thickening of the glomerular basement membrane, mesangial proliferation (Fig. 7) and increased fibrotic area (Table 3 and Fig. 7), with more evident effects after longer exposure (20 weeks).

## Discussion

Cardiovascular disorders together with kidney disease and diabetes are major health concerns with shared pathological mechanisms^10^. The risk of developing this combination of disorders is linked to aging, sedentary lifestyle and unhealthy dietary habits^13^. A recent advisory from the American Heart Association introduced the term CKM syndrome to describe and highlight this harmful interaction^13^. The global prevalence of CKM syndrome is rapidly rising, underscoring the need to better understand underlying pathogenic mechanisms and develop new preventive and therapeutic strategies to mitigate CVD morbidity and mortality.

Confronted with the necessity for preclinical models that account for the interplay of the three organs systems in CKM syndrome, our present study introduces and delineates a new murine model designed to replicate this multisystemic syndrome in a standardized manner. This model of CKM syndrome entails a surgical procedure involving the removal of 50% of available nephrons through UNX at an early age, coupled with a WD high in fat, sugar and salt. We show that mice with UNX and chronic consumption of this unhealthy diet developed kidney dysfunction, several features of metabolic syndrome and hypertension, progressively increasing the risk of CVD and related complications.

In CKM syndrome, each disease is not independent but affects and exacerbates the other systemic disorders. This arrangement of pathophysiological interactions, together with the integrated risk factors involved, requires an interdisciplinary medical approach and interventions that can simultaneously target multiple disorders^9,28^. There are known animal models reproducing each disease that contribute to CKM syndrome, such as for CKD^29–33^, T2D^34–37^ and CVD^38–41^. However, models available for preclinical studies of CKM syndrome, integrating all these rather complex pathological conditions, are scarce.

CKD and CVD often coexist and have been studied in various experimental models with mixed success in replicating clinical features. A systematic review and meta-analysis of murine models of uremic cardiomyopathy, together with a commentary on the same topic, aimed to identify suitable models for studying CKD-induced cardiac hypertrophy, fibrosis and function using ‘single-hit’ and ‘multifactorial-hit’ strategies^42,43^. It was concluded that genetic and cardiovascular risk factors are critical in developing uremic cardiomyopathy. When modeling CKD, 129/Sv mice, more than C57BL/6 mice, show significant cardiac hypertrophy, fibrosis and increased blood pressure. Multifactorial-hit models, incorporating these risk factors, offer a more reliable method for studying CKD-related cardiac pathologies than single-hit models and should be used in future research to enhance relevance and reproducibility^42,43^.

Funahashi and colleagues reviewed candidate animal models for acute or type 1 cardiorenal syndrome but without in-depth consideration of metabolic disorders^44^. Furthermore, the use of high-salt diets as an inducer of nondiabetic cardiorenal syndrome has also been explored^45^, with the aim of investigating the syndrome and interventions such as sodium–glucose cotransporter 2 inhibitors without simulating metabolic disturbance in T2D.

Our triple-hit model triggered the phenotypic alterations evident in metabolic syndrome and T2D, with the cardiovascular disorders predominantly attributed to the salt component of the diet, as previously documented in both rats^24^ and mice^46^. The blood pressure was increased by approximately 10 mmHg, and the thickening of cardiac wall suggests cardiac hypertrophy. Increased cardiomyocyte size in mice with UNX and WD compared with controls supports the development of cardiac hypertrophy, which progressed over time. Notably, the subsequent decrease in left ventricular FS seemed to progress over time, concurrently with worsened key metabolic indexes both at systemic and organ level. Given that systolic dysfunction is one of the hallmarks of diabetic cardiomyopathy, our model serves as a model of diabetic cardiomyopathy within the CKM syndrome complex^47^. Regarding the hepatic metabolism, interestingly, excessive salt consumption is reported to alter metabolic pathways in the liver^22,48^ and directly interfere with fat metabolism and obesity^21,49,50^, which is consistent with our current study.

The CKM syndrome model described in this study, despite no alteration in body weight gain, was associated with increased body fat accumulation and reduced lean mass. Analysis of adipose tissue showed increased gonadal and subcutaneous adipose tissue area, which was associated with decreased *Jam2* expression but unchanged expression of inflammatory markers in mice with UNX + WD (Table 1). Reduced vascularization of adipose tissue upon high-fat feeding is a well-established phenomenon, tightly correlated with the development of tissue hypertrophy and adipocyte enlargement^51–53^. Based on in-depth single-cell sequencing data and current literature^54^, *Jam2* expression is generally considered a reliable endothelial marker for adipose tissue vascularization. However, future studies could benefit from further immunohistochemical analyses to explore this phenomenon in more detail.

There is currently a high interest in the comorbidity-driven causes of heart failure with preserved ejection fraction (HFpEF), such as diabetes, hypertension and kidney disease^55^. These conditions are commonly associated with heart failure but are less understood in the context of HFpEF. It is well established that these comorbidities are critical risk factors for developing systolic dysfunction and heart failure with reduced ejection fraction (HFrEF). Hypertension alone is considered one of the most common causes of HFrEF^56^. The combination of diabetes, hypertension and kidney disease can lead to various cardiovascular phenotypes, including HFpEF or HFrEF.

A major research focus is identifying what determines whether patients with these shared comorbidities will develop HFpEF or HFrEF. In this context, our murine model is valuable as it uniquely combines three critical comorbidities (hypertension, kidney disease and metabolic syndrome) relevant to the development and progression of CVD. Based on our findings at 12 and 20 weeks follow-up (for example, reduced FS and increased troponin levels), our triple-hit model more closely represents HFrEF or heart failure with mildly reduced ejection fraction, rather than HFpEF.

Reduced NO bioavailability, often accompanied with oxidative stress, is considered a central theme in CVD and associated renal and metabolic comorbidities. Among the four markers of NO bioavailability measured in this study, plasma nitrite and heme–NO (which may be considered the best marker to evaluate NO bioactivity) were significantly decreased. In particular, the present model of CKM syndrome demonstrates a progressive decline in the red blood cell (RBC)-bound heme–NO levels. As heme–NO is an emerging nitric oxide synthase-related signaling species^57–59^, its decline may be of importance in CKM syndrome. Interestingly, the decline in heme–NO was paralleled with the development of endothelial dysfunction and impaired glucose tolerance. By contrast, liver DNIC levels tended to increase, which may be potentially regarded as a sign of inducible nitric oxide synthase (iNOS) induction and inflammation^60^.

Reduction of nephron number by UNX, after completed nephrogenesis at early age, results in a condition referred to as solitary functioning kidney (SFK). Under these circumstances, there is an acute volume overload and an elevated risk of injury due to glomerular hyperfiltration over time. Indeed, studies indicate the presence of kidney injury signs in up to 50% of children with SFK^61,62^. From a functional point of view, SFK initiates a cascade of adaptations to maintain vital functions.

In the short term, these structural modifications seem beneficial, but in the long term the adaptive processes of repair can become maladaptive. The mechanisms of this process are still being explored but may involve G2/M cell-cycle arrest, cell senescence, inflammation, profibrogenic cytokine production, and activation of pericytes and interstitial myofibroblasts^63,64^.

In the present model, UNX was responsible for volumetric stress and glomerular hyperfiltration to the remaining kidney; however, simultaneous exposure to a high-salt diet further encouraged renal tissue damage and reduced function, an accumulated effect most observed after 20 weeks of exposure and in line with the reported effects of high salt consumption on the body^48,65–67^.

The possible risks of excessive salt intake, seen over a long period, have been much discussed in recent decades. Based on experimental studies, high salt intake can negatively affect both kidney and cardiovascular function. It has been difficult to validate this observation in a clinical and populational perspectives^68,69^. However, a recently published study shows beneficial effects of reducing salt intake on blood pressure^70^. Reduction in dietary sodium significantly lowered blood pressure in the majority of middle-aged to older adults, which was independent of known hypertension and use of antihypertensive medication(s). Given the substantial impact of salt in our dietary habits and its potential contribution to the pathophysiological aspects of cardiovascular and renal disorders, such as CKD and CVD (including hypertension and heart failure), it is somewhat surprising that this specific dietary component has not been incorporated into the standard WD when investigating its adverse effects on metabolism in obesity and T2D.

There is an unmet medical need regarding prevention and treatment of cardiovascular complications in renal failure. Many contributing factors have been disclosed including hypertension, calcium–phosphate abnormalities, dyslipidemia and systemic inflammation, but no effective treatment has emerged yet. Statin treatment has not been shown to be efficient in advanced renal failure^71,72^. Anti-inflammatory treatment, using IL-6 blockade, has shown promising effects in smaller clinical trials in people with higher risk for CVD (for example, rheumatoid arthritis and renal failure)^73,74^, supporting the design of larger trials with a longer duration of follow-up.

A valid experimental model, in which mechanisms of disease could be studied and novel treatments could be tested on both short- and long-term basis and at a substantially lower cost compared with a clinical trial, is advantageous. However, some limitations must be considered in preclinical studies with laboratory animals. Even though many genes between human and model animals are highly similar in both sequence and function, their regulation and interplay can differ^75^.

During the experimental protocol of this novel CKM syndrome model, we achieved a 100% survival rate, even with a triple-hit pathological induction associated with substantial injuries in different organs and exposure for almost 5 months. When comparing groups exposed to the model for 12 and 20 weeks, a progression of the pathological phenotype was identified. Therefore, a longer approach may also be interesting to investigate the cumulative effects of longer exposure to the model, mortality rate and cause of death, as well as the possible characterization of end-stage renal disease and other terminal complications in the cardiovascular and metabolic systems.

## Conclusions

Facing the difficulty of experimentally reproducing the multisystemic disorders observed in CKM disorders, this study characterizes a new CKM syndrome model option for preclinical studies in rodents. In this model, an integrated approach was used by surgical removal of 50% of the nephrons (which largely reflects the decline in kidney function in middle-aged individuals), in combination with chronic feeding with a special high-salt-containing WD. This approach led to the development of kidney dysfunction, metabolic syndrome and hypertension, which progressed over time and increased the risk of CVD and associated complications. The described model shows clinically relevant features of CKM syndrome, with 100% survival of the experimental animals. We believe that this novel disease model holds promise for elucidating the pathophysiology of the development and progression of CKM syndrome and for exploring potential therapeutic strategies in future investigations.

## Methods

### Animals and experimental design

This study was approved (17816-2021) by the Regional Institutional Animal Care and Use Committee at Karolinska Institutet in Stockholm-Sweden and performed according to the National Institutes of Health guidelines and with the EU Directive 2010/63/EU for the conduct of experiments in animals. Commercially available conventional young male C57BL/6J mice (Janvier Labs) were housed in the animal facility of Comparative Medicine, Karolinska Institutet. They were maintained in conditions of controlled temperature, humidity and light–dark cycle (12/12 h), with ad libitum access to food and water.

A schematic of the experimental design is shown in Supplementary Fig. 1. The animals were brought to the facility at 21 days of age, and after 7 days of acclimatization, sham surgery or UNX was performed to remove 50% of the available nephrons (details described below) at 28 days of age. After 7 days and complete recovery from the surgery, the animals were randomly divided into three different groups: (1) sham surgery with regular rodent chow (RD, SDS DS801722G10R, CRM(P) 801722, SAFE); (2) UNX with the WD rich in sugar, fat and 4% salt (WD) tailor-made by Research Diets for 12 weeks; and (3) UNX with the same WD for a prolonged period of 20 weeks. Sample size (total number of mice) for the different groups were sham (*n* = 12); UNX + WD 12 weeks (*n* = 12) and UNX + WD 20 weeks (*n* = 8). Details on the composition of the experimental rodent chows are presented in Supplementary Table 1.

After starting the experimental protocol with dietary interventions (~35 days of age), the animals were monitored twice a week, and body weight, water and food consumption as well as other key biological parameters were recorded.

### UNX

After the acclimatization period, at 28 days of age, sham surgery or UNX was performed. For induction and maintenance of anesthesia, concentrations of isoflurane at ∼5% and 1.5% (vol/vol), respectively, were used (Pharmacia & Upjohn). The mice were placed on a heating table, and the abdominal cavity was accessed through the linea alba. The left kidney was located with a binocular stereomicroscope (Carl Zeiss Microscopy), and the renal artery and vein were ligated with 6–0 suture thread. Then, the ureter was ligated with the same suture thread and the kidney was dissected and removed from the abdominal cavity. After checking for bleeding, the abdominal muscles and skin were sutured separately. The mice were monitored until complete recovery from anesthesia, and the analgesia protocol was performed with buprenorphine twice a day until surgical recovery.

### Metabolic function

#### Body composition

Body composition was measured by dual-energy X-ray absorptiometry, using a Medikors InAnlyzer densitometer (MEDIKORS). The total amount of fat and lean mass in grams, the relative percentages compared with total mass, bone mineral density and bone mineral content were used as parameters to evaluate body composition.

#### Metabolic parameters

The metabolic parameters evaluated were plasma insulin, glucose concentrations in fasting (5 h) and nonfasting mice, intraperitoneal glucose tolerance test (ipGTT) and the skeletal muscle fibers glucose uptake ex vivo.

Mice were fasted for 5 h (morning fasting) before ipGTT. Blood glucose levels were measured by FreeStyle Lite Blood Glucose Meter (Abbott Diabetes Care) at 0, 15, 30, 60 and 120 min after intraperitoneal injection of 50% d-glucose solution (2 g/kg body weight).

Plasma insulin was analyzed by the Mouse Insulin ELISA Kit 10-1247-10 (Mercodia AB) according to the manufacturer’s instructions. The assay range was 0.2–6.5 µg/l, and the limit of detection was ≤0.2 µg/l. Both intraassay and interassay coefficients of the variations were ≤10%.

For the evaluation of glucose uptake in muscle fibers, skeletal muscles soleus and extensor digitorum longus were accurately isolated from mouse legs and kept in a relaxant solution: Krebs–Ringer–phosphate–Hepes (KRPH) buffer (20 mM 4-(2-hydroxyethyl)-1-piperazineethanesulfonic acid (Hepes), 5 mM KH_2_PO_4_, 1 mM MgSO_4_, 1 mM CaCl_2_, 136 mM NaCl and 4.7 mM KCl, pH 7.4) containing 2% bovine serum albumin (KRPHB), for 60 min at room temperature. Then, insulin 20 mUnits/ml was added and incubated at 30 °C for 20 min. After 20 min incubation of 1 mM 2-deoxyglucose at 30 °C, the muscles were washed with phosphate-buffered saline (PBS), and the glucose uptake assay was performed following the manufacturer’s instructions (Glucose Uptake Fluorometric Assay Kit, catalog number MAK084, Sigma-Aldrich).

#### Triglycerides and cholesterol

Triglycerides and cholesterol levels in plasma samples were analyzed by the Triglyceride Colorimetric Assay Kit (no. 10010303, Cayman Chemical) and Cholesterol Fluorometric Assay Kit (no. 10007640, Cayman Chemical), respectively. Plasma was diluted and protocols were performed according to the manufacturer’s instructions.

#### Fat tissue analysis

Gonadal white adipose tissue (gWAT) and subcutaneous white adipose tissue (scWAT) were collected upon termination. After rinsing with saline to remove furs, gWAT and scWAT were fixed with 4% paraformaldehyde for 48 h and then transferred to PBS and paraffin imbedded. All samples were cut into 5-μm-thick sections, stained with hematoxylin–eosin (HE) using standard protocols for paraffin-imbedded samples and imaged. gWAT and scWAT adipocyte size was measured as the cross-sectional area of 300 adipocytes per mouse and fat depot using the Adiposoft plugin of the Fiji ImageJ Software by analyzing 50 cells each from 6 random areas chosen in blind from each section, followed by manual inspection to ensure that only intact adipocytes were included by the semi-automatic software analysis. The inclusion range of adipocyte size was 30–120 µm for gWAT and 20–120 µm for scWAT to restrict the analysis to only adipocytes.

For transcriptional analyses of fat tissue, 20–30 mg of gWAT was homogenized in 1 ml Qiazol Lysis Reagent (Qiagen) using a TissueLyser and 5 mm stainless-steel beads (Qiagen) for 3 min at 30 Hz. The homogenates were centrifuged at 12,000*g* for 10 min at 4 °C, and the formed lipid layer was removed. RNA was extracted using the RNeasy Micro Kit and RNase-Free DNase Set (both Qiagen) and eluted in 30 μl RNase-free water. The RNA concentration was determined using Nanodrop, and reverse transcribed into cDNA using SuperScript II Reverse Transcriptase (Invitrogen). qPCR was performed using TaqMan Fast Advanced Master Mix, MicroAmp Fast Optical 96-well Reaction Plates (both Applied Biosystems) and TaqMan primers for *Cd68* (Mm03047343_m1), *Jam2* (Mm00470197_m1), *Pdgfra* (Mm00440701_m1), *Lipe* (Mm00495359_m1), *Lpl* (Mm00434764_m1), *Ptprc* (Mm01293577_m1), *Il6* (Mm00446190_m1), *Ccl2* (Mm00441242_m1), *Il1b* (Mm00434228_m1) and *Tbp* (Mm00446971_m1).

### Cardiovascular function

#### Blood pressure

Coda High Throughput Noninvasive Tail Monitoring System (Kent Scientific) was used for conscious blood pressure monitoring, according to the manufacturer’s protocol. The system uses volume pressure recording to measure the blood pressure by detecting the tail blood volume. Systolic arterial pressure, diastolic arterial pressure and MAP were determined by 45 repetitions. Averaged data from each animal were used for analysis.

#### Echocardiography

Cardiac function was noninvasively evaluated by transthoracic echocardiography using the Philips HDI 5000 imaging system as previously reported^76–78^. Briefly, the mice were anesthetized with gas mixture of oxygen and isoflurane (2–3%), and echocardiograms were obtained with a 7–15 MHz CL 15–7 scanning head to perform left ventricle short-axis echo M-mode imaging. Heart contractility was measured as percentage of left ventricular FS using the following formula: LVd − LVs/LVd × 100, where LVd and LVs stand for left ventricle diastole and systole dimensions, respectively. Left ventricular wall thickness was assessed by measuring IVS and posterior wall dimension.

#### Vascular reactivity

After removing adipose and connective tissues surrounding mesenteric arteries, the vessel was segmented into approximately 2 mm segments. The segments were then mounted on wire myograph system (Model 620M; Danish Myo Technology) using 25 µm wire, which is connected to Powerlab system (Powerlab 4/30) to record the isometric tension. The chambers were prefilled with 8 ml of physiological salt solution (37 °C, pH 7.4) aerated with carbogen (95% O_2_; 5% CO_2_). After mounting, a loading force of 2.5 mN was applied to the vessel to mimic the near-physiological pressure, and the vessels were equilibrated for 45 min. Thereafter, vessel segments were contracted with 75 mM KCl together with 10 μM phenylephrine (PE) to determine the maximum contractive reactivity of the vascular smooth muscle cells. Vessels were then washed three times and normalized for 40 min before obtaining each concentration–response curve for PE (0.1 nM to 10 μM), Ach (0.1 nM to 100 μM) and SNP (0.1 nM to 10 μM). Initial contraction, at approximately 70% of maximum contraction, was induced by PE before application of Ach or SNP to observe endothelial-dependent or endothelial-independent vasorelaxation, respectively.

#### Mitochondrial function

Kidney and heart mitochondria were isolated by differential centrifugation, as described elsewhere^79^. Mitochondrial function was evaluated using high-resolution respirometry (Oroboros, O2k). Preserved mitochondrial integrity was confirmed by measuring respiratory control ratio defined as maximal complex I-mediated maximal respiratory capacity (pyruvate (5 mM), malate (2 mM) and ADP (2.5 mM)) divided by leak state without adenylates (pyruvate and malate).

Maximal complex I (CI)-dependent respiration was evaluated in the presence of pyruvate (5 mM), malate (2 mM) and ADP (2.5 mM) and complex II (CII)-dependent respiration in the presence of succinate (10 mM), ADP (2.5 mM) and rotenone (0.5 μM). Maximal CI + CII-dependent respiration was measured in the presence of pyruvate, malate, succinate and ADP. Maximal complex IV (CIV) activity was evaluated by adding ascorbate (2 mM), *N*,*N*,*N*′,*N*′-tetramethyl-*p*-phenylenediamine (TMPD) (0.5 mM) in presence of antimycin (2.5 μM), ADP (2.5 mM) and cytochrome *c* (10 μM). Correction was made for autooxidation of TMPD and ascorbate at the corresponding oxygen tension. Leak respiration was measured in the presence of pyruvate (5 mM), malate (2 mM) and oligomycin (2.5 μM). Respiration was normalized to mitochondrial protein or tissue wet weight. Mitochondrial H_2_O_2_ production was measured spectrofluorometrically using the amplex red system (5 μM, Amplex ultrared) together with horseradish peroxidase (1 U/ml) (Sigma-Aldrich P 8250). Calibration of the H_2_O_2_ signal before each experiment was performed by adding standard solution of hydrogen peroxide (180 nM). Mitochondrial oxygen efficiency (P/O ratio) was determined in presence of CI substrates and ATP (2 mM) by steady-state infusion of nonsaturating levels of ADP using the titration-injection micropump (TIP2k) for two-channel operation. The number of mitochondria in the chamber was adjusted so that respiration during ADP infusion corresponded to approximately 50% of maximal state 3 respiration. The P/O ratio was calculated as the rate of infused ADP divided by the oxygen consumed at steady-state respiration. Background correction was performed for the infused oxygen present in the ADP solution.

#### Heart tissue qPCR analysis

Approximately 20 mg of snap-frozen pieces of the heart were homogenized in 1 ml TRIzol RNA isolation reagents (15596026, Thermo Fisher Scientific) by tissue lyser for total RNA extraction. After phase separation achieved by chloroform, the RNA-containing aqueous phase was transferred to a new tube, precipitated by isopropanol and washed with 70% ethanol. Purified total RNA was then eluted in RNAse-free water. The concentration of the obtained RNA was determined in a NANODROP 1000 spectrophotometer (Thermo Scientific). Subsequently, reverse transcription was conducted from 2.5 μg of the total RNA with SuperScript III First-Strand Synthesis System (18080-051, Invitrogen) according to the manufacturer’s instructions. To detect DNA amplification in qPCR reaction, SYBR green master mix (BIO-RAD) was used on a CFX 384 Real Time System (Bio-Rad) for the following genes: *Nppa* (NM_008725.3), *Nppb* (NM_008726.5), *Postn* (NM_001368678.1), *Myh7* (NM_001425737.1), Myh6 (NM_001164171.1), *Il6* (NM_001314054.1), *Il1b* (NM_008361.4) and *Gapdh* (NM_001289726.1). Primer sequences are available upon request. The thermal cycling condition was initiated by denaturation at 95 °C for 3 min, followed by 40 cycles of 95 °C for 10 s and 60 °C for 30 s. The ΔΔCt method was used to analyze the changes in gene expression relative to the sham group.

#### Inflammatory and cardiac injury biomarkers

As a marker of direct myocardial injury, the levels of cardiac troponin I were quantified in plasma samples using the colorimetric sandwich ELISA assay (no. NBP3-00456, Novous Biologicals). IL-6 was quantified as an inflammatory marker by the IL-6 Mouse ProQuantum Immunoassay Kit (catalog no. A43656, Invitrogen). Procedures and dilutions were performed according to the manufacturer’s instructions.

#### Electron paramagnetic resonance

The levels of heme–NO (RBC) and DNIC (whole liver) were measured by electron paramagnetic resonance (EPR) using an X-band table-top spectrometer MS5000 (Bruker-Magnettech). All samples were prepared within 10 min after extraction from the animals and were frozen and kept in liquid nitrogen. The EPR spectra were recorded at 77 K, and the instrument parameters were 10 mW microwave power, 0.6 mT amplitude modulation, 100 kHz modulation frequency, 330 mT center field, 40 mT sweep width, 60 s sweep time and 4 scans. The RBC heme–NO levels were assessed by measuring the first component of the characteristic triplet EPR signal at *g* value (a spectroscopic tensor) of 2.01 and aN (superfine electron splitting at N-atom) of 1.7 mT. The DNIC levels were estimated by measuring the EPR feature at *g* value = 2.04 (ref. ^80^). The EPR data are presented in arbitrary units (a.u.).

### Kidney function

#### GFR

The GFR was determined using clearance kinetics of plasma fluorescein isothiocyanate-inulin (FITC-inulin). A 1% solution of FITC–inulin (Sigma F3272) in PBS was filter-sterilized by a 0.22 μm filter. Approximately 100 µl of the solution was injected into the tail vein of the animals, and blood samples were taken in heparinized capillaries at 1, 3, 5, 10, 15, 35, 55 and 75 min after the injection. Blood samples were centrifuged to obtain plasma and buffered to PBS pH 7.4 with 500 mM Hepes. Fluorescence intensity was determined by multi-mode microplate readers (SpectraMax iD3) with excitation at 480 nm and emission at 530 nm. The linear correlation between fluorescence intensity and inulin concentration was established by a standard curve generated by testing known FITC–inulin concentrations.

#### Plasma and urine biomarkers

Plasma and urinary creatinine were measured by creatinine colorimetric assay kits (no. 700460, Cayman Chemical and no. 500701, Cayman Chemical, respectively). Urinary albumin level, a well-recognized biomarker for early-stage CKDs, was analyzed using a fluorometric assay kit (no. ab241017, Abcam). Urea in plasma was detected by a colorimetric detection kit, BUN (no. EIABUN, Invitrogen, ThermoFisher). Urine-specific gravity was measured by refractometry, employing an analog refractometer (KERN ORA-P). All procedures and dilutions were performed according to the manufacturer’s instructions. Plasma and urinary nitrate and nitrite were analyzed by high-performance liquid chromatography (ENO-20; EiCOM) as described previously^81^. In brief, nitrite and nitrate were separated by reverse-phase ion-exchange chromatography followed by nitrate reduction to nitrite by cadmium and reduced copper. Griess reagent was then used for derivatization of reduced nitrite to form diazo compounds that can be detected by a visible detector at 540 nm.

### Histopathological evaluation

Kidney, liver, heart and duodenum were collected and fixed with 4% paraformaldehyde for histopathological analysis. After fixation, the samples were embedded in paraffin and sectioned into 5 µm. The kidney slides were stained with HE, PAS and PicroSirius Red (PSR). Liver and duodenum slides were stained with only HE, and heart slides was stained with HE and PSR to highlight collagen fiber content. All slides were blindly evaluated by a specialized histopathologist.

Tissue morphology such as fibrosis, necrosis and inflammatory infiltrate were investigated and quantitated. Hepatic fat deposition was evaluated by the percentage of fat area in five random fields per animal (20× objective). For the evaluation of the intestinal absorption area, the villi length and the crypts depth of duodenum were measured in five random fields per animal (10× objective). The villus:crypt ratio was used as an index.

To depict the tubular damage in kidney, a scoring system, derived from the distribution of previously described alterations such as tubular vacuolization, dilatation and atrophy, loss of brusher border and thickening of tubular basement membrane, was established^18^.

The glomerular injury was determined on the basis of glomerular dilation, glomerulosclerosis, mesangial hypercellularity and matrix expansion and irregular thickening of the glomerular basement membrane in eight random fields per sample. The percentage of fibrotic area was evaluated with PSR-stained slides and quantified as the areas with positive red staining in the renal cortex in relation to the total area evaluated.

For the heart, interstitial and perivascular fibrosis were quantified from PSR-stained slides. Bright-field images covering the cardiac cross-sectional area were captured at ×20 and ×40 magnifications. Collagen-positive staining was quantified by applying the particle analysis command in ImageJ software and determining the percentage of the positive area. Interstitial fibrosis was quantified as a percentage of the total tissue area, while perivascular fibrosis was quantified as a percentage of the perivascular tissue area. The cardiomyocytes cross-sectional size was automated quantified using the CmyoSize plugin available for the ImageJ software. All images were captured by Axioscope Microscope and Camera Axiocam 208 color (Carl Zeiss Microscopy) and quantified in Fiji/ImageJ Software 1.54 version.

### Statistics

Data were presented as mean ± standard deviation (s.d.) unless otherwise indicated. Statistical analysis was performed in GraphPad Prism version 9.2.0 (GraphPad Software) by applying one-way or two-way ANOVA, followed by Tukey’s multiple comparisons test for comparisons among the groups. Comparison between two groups was assessed by unpaired *t*-test. *P* < 0.05 was considered statistically significant.

### Reporting summary

Further information on research design is available in the Nature Portfolio Reporting Summary linked to this article.

## Online content

Any methods, additional references, Nature Portfolio reporting summaries, source data, extended data, supplementary information, acknowledgements, peer review information; details of author contributions and competing interests; and statements of data and code availability are available at 10.1038/s41684-024-01457-5.

## Supplementary information

Supplementary Information Supplementary Fig. 1 and Tables 1 and 2.

Reporting Summary

## Source data

Source Data Fig. 1 Raw data of metabolic parameters.

Source Data Fig. 3 Raw data of cardiovascular functional parameters.

Source Data Fig. 4 Raw data of cardiovascular biochemical and transcriptional parameters.

Source Data Fig. 5 Raw data from histopathological evaluation of the heart.

Source Data Fig. 6 Raw data of renal parameters.

## Data Availability

The data that support the findings of this study are available from the corresponding author upon request. Source data are provided with this paper.
