# Supplementary material for: A novel model of cardiovascular–kidney–metabolic syndrome combining unilateral nephrectomy and high-salt–sugar–fat diet in mice
Source: Lab Anim (NY). 2024 Oct 22;53(11):336–46. doi: 10.1038/s41684-024-01457-5 (PMC11519006; doi:10.1038/s41684-024-01457-5)
Supplement: Supplementary file 1 — Supplementary Fig. 1 and Tables 1 and 2. [file 41684_2024_1457_MOESM1_ESM.pdf]

lab animal

Supplementary information

https://doi.org/10.1038/s41684-024-01457-5

A novel model of cardiovascular–kidney–metabolic syndrome combining unilateral nephrectomy and high-salt–sugar–fat diet in mice

In the format provided by the authors and unedited

Table of Contents

Supplementary Figure 1. Schematic of experimental design for a model of cardiovascular-kidney-metabolic (CKM) syndrome in mice……………………………………………………………………..……………………**2**

Supplementary Table 1. Composition of special Western diet…………………………………………………….**3**

Supplementary Table 2. Description of sample numbers……………………………………………………………**4**

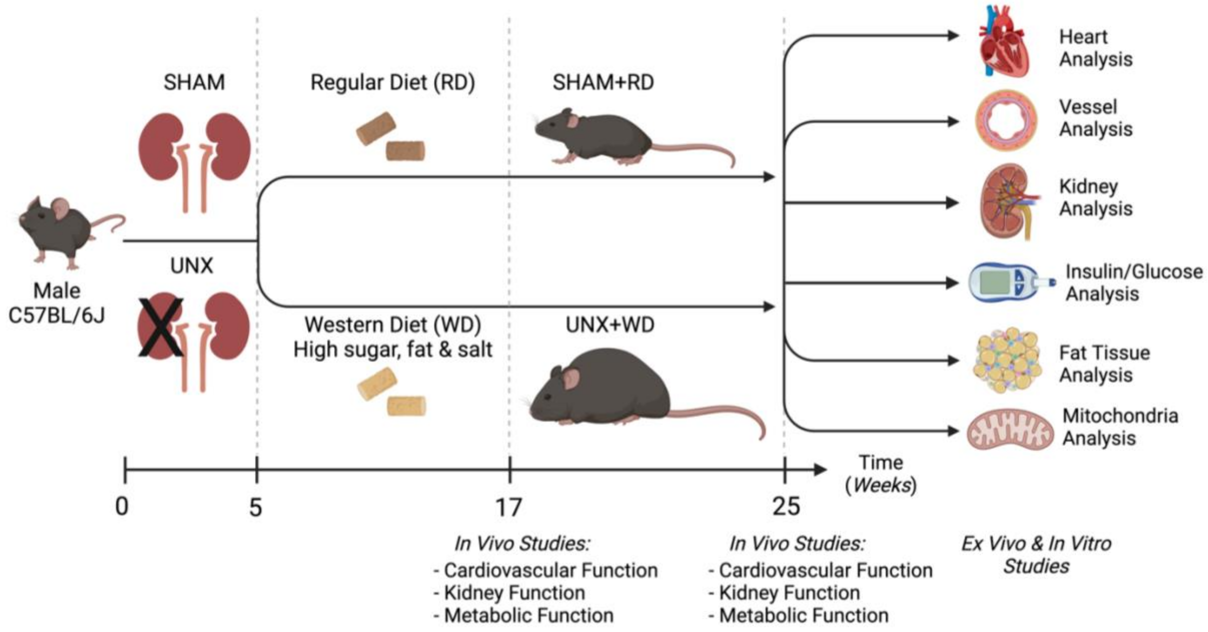

Supplementary Figure 1: **Schematic of experimental design for a model of cardiovascular-kidney-metabolic (CKM) syndrome in mice.** Male C57BL/6J mice (4 weeks old) underwent unilateral nephrectomy (UNX) and were subsequently fed a customized Western diet (WD) high in sugar, fat, and salt for 12 or 20 weeks. Sham-operated mice, fed regular rodent diet for the same durations, served as healthy controls (SHAM+RD). *In vivo* studies assessed cardiovascular function (blood pressure and echocardiography), kidney function (glomerular filtration rate), and metabolic functions (body composition, weight gain, glucose metabolism, fat distribution). These *in vivo* assessments were followed by *ex vivo* functional and *in vitro* mechanistic studies, which included isolated vessel reactivity, mitochondrial respiration, histopathology, and biochemical analysis of various tissues. Illustration created with BioRender: Carlstrom, M. (2024) BioRender.com/s35e756.

Supplementary Table 1. Composition of special Western diet.

Class description	Ingredients	Grams (g)
Protein	Casein, Lactic, 30 Mesh	200.00
Protein	Methionine, DL	3.00
Carbohydrate	Sucrose, Fine Granulated	500.00
Carbohydrate	Starch, Corn	150.00
Fiber	Solka Floc, FCC200	50.00
Fat	Corn Oil	50.00
**Mineral**	**Sodium Chloride**	**42.00**
Mineral	S10001	35.00
Vitamin	V10001	10.00
Vitamin	Choline Bitartrate	2.00
Dye	Dye, Green FD&C #40	0.05
**Total**		**1042.05 g**

Detailed formulation of the customized Western diet rich in sugar, fat, and salt (WD) produced by Research Diets, Inc. (New Brunswick, NJ, 08901 USA), Product code D06111702-1.5Vii. This product is a modified version of Research Diet's traditional WD (D12079B-1.5Vii).

Supplementary Table 2. **Description of sample numbers.**

	SHAM+RD	UNX+WD 12 weeks	UNX+WD 20 weeks
**Fig 1A**	8	7	8
**Fig 1B**	12	12	8
**Fig 1C**	12	12	6
**Fig 1D**	12	12	8
**Fig 1E**	12	4	8
**Fig 1F**	12	4	8
**Fig 3A**	12	12	8
**Fig 3B**	12	8	8
**Fig 3C**	12	8	8
**Fig 3D**	8	8	8
**Fig 3E**	8	8	8
**Fig 3F**	8	8	8
**Fig 3G**	11	7	5
**Fig 3H**	10	8	5
**Fig 3I**	12	12	8
**Fig 4A**	6	7	8
**Fig 4B**	6	7	8
**Fig 4C**	6	7	8
**Fig 4D**	12	12	8
**Fig 4E**	9	10	6
**Fig 4F**	6	7	8
**Fig 5A**	8	8	8
**Fig 5B**	12	12	8
**Fig 5C**	40*	41*	39*
**Fig 6A**	12	12	8
**Fig 6B**	12	12	8
**Fig 6C**	11	11	7
**Fig 6D**	11	8	6

Description of sample numbers by methods used in figures 1, 3, 4, 5 and 6. *Number of photomicrographs analyzed - average of 4 photomicrographs per tissue sample. Abbreviations: RD, regular diet; UNX, unilateral nephrectomy; WD, Western diet.
